# Self-reported oral and general health in relation to socioeconomic position

**DOI:** 10.1186/s12889-017-4609-9

**Published:** 2017-07-26

**Authors:** Magnus Hakeberg, Ulla Wide Boman

**Affiliations:** Department of Behavioral and Community Dentistry, Institute of Odontology, The Sahlgrenska Academy, University of Gothenburg, P.O. Box 450, 40530 Gothenburg, SE Sweden

**Keywords:** Socioeconomic status, Health, Oral health, Survey

## Abstract

**Background:**

During the past two decades, several scientific publications from different countries have shown how oral health in the population varies with social determinants. The aim of the present study was to explore the relationship between self-reported oral and general health in relation to different measures of socioeconomic position.

**Methods:**

Data were collected from a randomly selected sample of the adult population in Sweden (*n* = 3500, mean age 53.4 years, 53.1% women). The response rate was 49.7%. Subjects were interviewed by telephone, using a questionnaire including items on self-reported oral and general health, socioeconomic position and lifestyle.

**Results:**

A significant gradient was found for both oral and general health: the lower the socioeconomic position, the poorer the health. Socioeconomic position and, above all, economic measures were strongly associated with general health (OR 3.95) and with oral health (OR 1.76) if having an income below SEK 200,000 per year. Similar results were found in multivariate analyses controlling for age, gender and lifestyle variables.

**Conclusions:**

For adults, there are clear socioeconomic gradients in self-reported oral and general health, irrespective of different socioeconomic measures. Action is needed to ensure greater equity of oral and general health.

## Background

During the past two decades, several scientific publications from different countries have shown how oral health in the population varies with social determinants [1–13]. The measures of oral health have been different; both subjective and objective variables have been used with somewhat diverse outcomes relative to the covariates under study. Importantly, gradients of oral health with respect to socioeconomic position have been revealed, and a similarity with general health has also been found [5]. Sabbah et al. [5] showed in their study that self-reported levels of oral and general health differed depending on income and education. Thus, the lower the income and educational level, the higher the probability of reporting poor oral and general health. Moreover, these results also applied to objective measures such as periodontitis and ischaemic heart disease. Luchi et al. [4] used self-rated oral health when analysing a Brazilian cohort with different socioeconomic positions. The results revealed that several of the socioeconomic variables were highly significant and displayed an oral health gradient, showing that the lower the income and educational level, the greater the proportion of individuals with poor oral health. Similar results have been reported in Western Europe and Scandinavia [7, 10, 14, 15]. Ståhlnacke et al. [7] from Sweden used an index, ‘perceived oral health’, to analyse changes with regard to social gradients over a five-year period. The authors found significant associations between gradients in education and occupation; the lower the educational level the poorer the perceived oral health. The estimated correlations were stable over time. In a recent publication Molarius et al. [13] report from a Swedish sample concerning socioeconomic status and oral health. The authors found an association between self-rated poor oral health and unemployment, being foreign-born and having no cash margin. There is a lack of knowledge of how self-reported general, oral health and socioeconomic measures are related. Moreover, few studies have used national random samples of adult individuals to analyse the above relationship. The aim of this study was to model self-reported health on different socioeconomic measures, such as income and education, in an attempt to reveal possible inequalities in health and the magnitude of the differences in inequalities among adults.

## Methods

The present survey included a random sample of the adult population in Sweden. A telemarketing company, TNS SIFO, which is a Swedish company that performs public opinion and market surveys, was responsible for the sample selection and the interviews with the individuals by telephone. The participants were randomly selected from the SPAR register in Sweden. The SPAR (Swedish Personal Address Register) includes all individuals who are registered as being resident in Sweden. The exclusion criterion was individuals who did not speak and/or understand Swedish. The number of adults (aged ≥19 years) included in the sample was *n* = 3500, giving a participation rate of 49.7%. Due to the non-response of the non-participants, a comparison between participants vs. non-participants was not possible to conduct. However, when comparing with official data for the general Swedish population (Statistics Sweden, www.scb.se) certain differences were found (age, gender and ethnicity). Participants in the survey were somewhat older (53 vs. 49 years of age), proportionally more women were included in the study (53.1% vs. 50.5%) and fewer foreign-born (10.1% vs. 18.0%). The Regional Ethical Review Board in Gothenburg, Sweden approved of the study (Reg. No. 801–12).

A questionnaire with items concerning demography, self-rated oral and general health, education, income, dental care behaviour, and lifestyle issues was used in the study. There were a total of 39 questions; however, the questions pertaining to self-rated health and socioeconomic position (SEP) were the focus of the analysis. The following variables were used in the analysis:

(i) Self-perceived health: How good is your general health? (five response options: poor (1), fair, good, very good, excellent (5)). The scale was dichotomised into poor/fair (Poor) vs. good/very good/excellent (Good); How good is your oral health? (four response alternatives: poor (1), fair, good, very good (4). The scale was dichotomised into poor/fair (Poor) vs. good/very good (Good).
(ii) SEP variables, such as gender, marital status (married/cohabiting vs. single), income in SEK (<200,000, 201,000–400,000, 401,000–600,000, ≥ 601,000) for the whole household (SEK 10 ~ 1 euro), level of education (primary school, high school, university, Masters or PhD degree), however in the multivariate analyses the categories university and Masters or PhD degree were collapsed into one category (university), estimated financial resources for unforeseen expenditure (SEK 15,000 in one week, four response alternatives: yes, always; yes, mostly; no, mostly not; no, never), however in the multivariate analyses the categories “no mostly not” and “no never” were merged into one category. Ethnicity was categorised as foreign-born or Swedish-born, including the Nordic countries, and housing as rented flat, owner-occupied flat, or own house.
(iii) Lifestyle variables: How much do you exercise (physical activity) during your leisure time? (five response alternatives: no exercise (1), a little, once a week, twice a week or more, intensive exercise at least twice a week (5)); smoking (yes, previous smoker, no).

The descriptive analysis included frequencies, measures of central tendency (means), and variability (standard deviation). Bivariate analyses were performed using the t test, the Mann–Whitney test, the chi-square test, and logistic regression. Multivariate logistic regression analysis (MLR) was used with the self-rated health variables as the dependent variables and the socioeconomic indicators as the independent variables. Two different models were applied to the MLR; Model 1 included age and gender as covariates, and in Model 2, the analysis also included the lifestyle factors of smoking and physical activity. Model evaluations were performed using the Hosmer-Lemeshow and Nagelkerke test statistics. The pre-selected level of significance was α = 0.05. Due to some missing responses, the numbers of observations differ in the analysis. Thus, missing answers in the presented variables range between 0 and 52, except for the variable of income, where *N* = 502 answers are missing. When multiple comparisons were performed, Bonferroni corrections were applied.

## Results

Tables 1 and 2 show descriptive data for SEP, self-reported and lifestyle variables. The SEP variables showed differences between genders with women reporting higher educational levels, but men having a higher income and greater ability to obtain a large sum of money in one week. With regard to self-reported health, women, interestingly, scored somewhat higher levels on oral health but significantly poorer general health than men.

**Table 1:** Descriptive statistics for the included measures of socioeconomic position

Variable	Items	Women	Men	Total
Age (years) *N* = 3500		53.9 (17.6)	52.8 (17.4)	53.4 (17.5)
Gender *N* = 3500		53.1	46.9	
Marital status* *N* = 3495	Married/cohabiting Single	70.6 29.4	75.5 24.5	72.9 27.1
Education* *N* = 3478	Primary High school University Masters/PhD	17.2 36.8 45.1 0.9	19.1 44.1 35.7 1.2	18.1 40.2 40.7 1.0
Income (SEK)* *N* = 2998	<200,000 201,000–400,000 401,000–600,000 ≥601,000	20.8 31.5 25.6 22.0	13.3 26.0 27.8 32.8	17.1 28.8 26.7 27.4
Ability to obtain SEK 15,000 in one week.* *N* = 3448	No, never No, mostly not Yes, mostly Yes, always	9.1 8.7 32.6 49.6	5.9 5.4 26.4 62.4	7.6 7.1 29.7 55.6
Housing *N* = 3490	Rented flat Owner-occupied flat Own house	25.3 18.5 56.2	22.8 18.0 59.3	24.1 18.3 57.7
Ethnicity *N* = 3497	Swedish-born (+ Nordic countries) Foreign-born	91.7 8.3	92.3 7.7	91.9 8.1

Mean and standard deviation (age) and proportions for other variables. Asterisk (*) for statistically significant differences between women and men (*p* < 0.05)

**Table 2:** Descriptive statistics for the included measures of health and life style (proportions)

Variable	Item	Women	Men	Total
Oral health *N* = 3490	Poor Fair Good Very good	3.0 22.7 44.6 29.6	3.3 25.4 43.7 27.6	3.2 24.0 44.2 28.7
General health* *N* = 3489	Poor Fair Good Very good Excellent	3.7 13.6 33.3 35.1 14.4	1.5 10.3 33.8 35.1 19.1	2.7 12.0 33.5 35.1 16.7
Physical activity *N* = 3490	No A little Once/week ≥Twice/week Intensive ≥Twice/week	7.3 15.6 8.8 21.6 46.7	8.6 17.3 9.5 20.9 43.7	7.9 16.4 9.2 21.3 45.3
Smoking* *N* = 3496	Yes Previous smoker No	9.2 29.5 61.3	9.0 33.8 57.2	9.1 31.5 59.4

* = statistically significant differences between women and men (*p* < 0.05)

The proportions of poor health relative to the SEP variables are shown in Tables 3 and 4. For all variables there are significant gradients, where the lower the SEP, the poorer the health. Moreover, the proportions of poor self-reported oral health seem to be higher than the proportions for general health, except for the ‘income’ variable, for which the opposite result was found. For example, the educational category ‘Masters or PhD degree’ revealed that 19.4% of the participants reported poor oral health, while none in that category experienced poor general health. The opposite result was found for the highest income category, which showed 5% with poor oral health and 19% with poor general health.

**Table 3:** Self-rated oral and general health (poor vs. good) by education and income in SEK (*N* = number of individuals; % with poor health)

Education				
Oral health*	Primary school	High School	University	Masters or PhD
N	625	1395	1413	36
% with poor health	37.4	27.5	22.3	19.4
General health*	Primary school	High school	University	Masters or PhD
N	624	1395	1413	36
% with poor health	27.2	15.1	9.2	0.0
Income				
Oral health*	≤200,000	201,000–400,000	401,000–600,000	>600,000
N	510	861	798	821
% with poor health	26.2	18.3	10.1	5.0
General health*	≤200,000	201,000–400,000	401,000–600,000	>600,000
N	510	861	798	821
% with poor health	32.9	32.6	26.4	19.0

Chi-square test for linear trend, * = *p* < 0.05

**Table 4:** Self-rated oral and general health (poor vs. good) by housing, and ability to obtain SEK 15,000 in one week, (*N* = number of individuals; % with poor health)

Housing				
Oral health*		Rented flat	Owner-occupied flat	Own house
N		271	162	510
% with poor health		32.4	25.4	25.4
General health*		Rented flat	Owner-occupied flat	Own house
N		169	103	239
% with poor health		20.2	16.2	11.9
Obtain SEK 15,000 in one week				
Oral health*	No, never	No, mostly not	Yes, mostly	Yes, always
N	127	88	290	428
% with poor health	49.2	35.9	28.4	22.4
General health*	No, never	No, mostly not	Yes, mostly	Yes, always
N	83	48	164	205
% with poor health	31.9	19.5	16.1	10.7

Chi-square test for linear trend, * = *p* < 0.05

Obvious patterns were revealed (Table 5) when the health variables vs. the SEP variables were analysed in bivariate logistic regressions. Independently of general or oral health, a typical gradient of increasing odds ratios was found: the lower the educational level, the lower the income level or the possibility of obtaining a substantial amount of money in one week, or the poorer the housing situation, the higher the likelihood of reporting poor health*.* Another pattern in the data is that the odds ratios are of a higher magnitude in all categories for poor general health than for poor oral health. The SEP variable showing the steepest gradient is the income measure with an odds ratio of 3.95, indicating an almost four times higher risk of reporting poor general health if having an income below SEK 200,000 per year. For poor oral health, the highest odds ratio (2.61) was found when individuals reported no or little possibility of obtaining a sum of SEK 15,000 in one week.

**Table 5:** Bivariate logistic regression with oral and general health as the dependent variable (good vs. bad health), respectively, and socioeconomic position as the independent variable

		Oral health		General health	
		OR	95% CI	OR	95% CI
Education	University (Reference)	1.0		1.0	
	High school	1.33	1.12–1.58	1.80	1.43–2.27
	Primary	2.10	1.71–2.57	3.80	2.95–4.89
Income	≥601,000 (Reference)	1.0		1.0	
	401,000–600,000	1.53	1.21–1.94	2.14	1.45–3.16
	201,000–400,000	2.06	1.65–2.59	4.27	2.98–6.11
	≤200,000	2.09	1.63–2.70	6.75	4.66–9.78
Obtain SEK 15,000 in one week	Yes, always (Reference)	1.0		1.0	
	Yes, mostly	1.38	1.16–1.64	1.59	1.28–1.99
	No, mostly not/no never	2.59	2.11–3.19	2.91	2.27–3.72
Housing	Own house (Reference)	1.0		1.0	
	Owner-occupied flat	1.00	0.82–1.23	1.43	1.11–1.84
	Rented flat	1.43	1.20–1.72	1.87	1.50–2.34

The multivariable models are shown in Tables 6 and 7 for general and oral health, respectively. In Model 1, the SEP variables were adjusted for age and gender and in Model 2, the lifestyle variables of physical activity and smoking were added to the model. For general health, all four SEP variables showed the same gradient; i.e., the worse off a person is with regard to education, income, ability obtain a large sum of money at short notice, and housing, the greater the likelihood of reporting poor health over and above the other independent variables of age, gender, physical activity and smoking (Table 6). In Model 2, the odds ratios for all SEP variables and categories were attenuated compared with Model 1, while the overall model estimator (Nagelkerke) showed improvement from around 0.10 up to 0.20 in Models 1 and 2, respectively. Parallel results were found for the dependent variable oral health in Models 1 and 2, respectively, albeit the odds ratios being smaller and having a less steep gradient compared with the general health models (Table 7). In the full models, the variables marital status (OR (95%CI): 0.82 (0.62–1.09) and 1.03 (0.83–1.29)) and ethnicity (OR (95%CI): 0.98 (0.63–1.51) and 1.14 (0.84–1.56)) for general health and oral health, respectively, were not found to be significant predictors and did not improve the models.

**Table 6:** Multiple logistic regression Models 1 and 2 with general health as the dependent variable (good vs. bad)

		Model 1		Model 2	
		OR	95% CI	OR	95% CI
Education	University (Reference)	1.0		1.0	
	High school	1.91	1.50–2.42	1.64	1.28–2.09
	Primary	2.42	1.85–3.17	2.02	1.53–2.66
Income	≥601,000 (Reference)	1.0		1.0	
	401,000–600,000	1.92	1.29–2.84	1.66	1.11–2.47
	201,000–400,000	3.04	2.10–4.40	2.60	1.79–3.79
	≤200,000	4.73	3.21–6.97	3.95	2.66–5.88
Obtain SEK 15,000 in one week	Yes, always (Reference)	1.0		1.0	
	Yes, mostly	1.80	1.43–2.27	1.73	1.37–2.20
	No, mostly not/no never	3.51	2.69–4.56	3.11	2.36–4.10
Housing	Own house (Reference)	1.0		1.0	
	Owner-occupied flat	1.37	1.05–1.77	1.38	1.06–1.80
	Rented flat	2.24	1.78–2.83	2.11	1.66–2.67

Model 1 includes age and gender as covariates, while in Model 2, the lifestyle factors of smoking and physical activity were added to the model

**Table 7:** Multiple logistic regression Models 1 and 2 with oral health as the dependent variable (good vs. bad)

		Model 1		Model 2	
		OR	95% CI	OR	95% CI
Education	University (Reference)	1.0		1.0	
	High school	1.32	1.11–1.57	1.20	1.01–1.43
	Primary	1.71	1.38–2.13	1.49	1.18–1.84
Income	≥601,000 (Reference)	1.0		1.0	
	401,000–600,000	1.52	1.20–1.93	1.40	1.10–1.78
	201,000–400,000	1.94	1.54–2.44	1.73	1.37–2.19
	≤200,000	2.00	1.54–2.60	1.76	1.35–2.30
Obtain SEK 15,000 in one week	Yes, always (Reference)	1.0		1.0	
	Yes, mostly	1.53	1.28–1.82	1.46	1.22–1.75
	No, mostly not/no never	3.02	2.44–3.74	2.61	2.10–3.26
Housing	Own house (Reference)	1.0		1.0	
	Owner-occupied flat	1.01	0.82–1.24	0.98	0.79–1.21
	Rented flat	1.61	1.34–1.93	1.48	1.23–1.79

Model 1 includes age and gender as covariates, while in Model 2, the lifestyle factors of smoking and physical activity were added to the model

## Discussion

This cross-sectional epidemiological survey presents data from Sweden analysing how self-reported general and oral health are associated with different types of socioeconomic measures. The sample consisted of randomly chosen adult individuals who were asked to answer a battery of questions during a telephone interview. The main findings were that all SEP variables—education, income, the possibility of obtaining a given sum of money in one week, and the participants’ type of housing—had (i) a similar strength of association with the respective outcome, and (ii) showed a gradient relationship with the outcome. The lower the educational level, the lower the income, the smaller the chance of obtaining a large sum of money, and the poorer the housing situation, the higher the risk of reporting poor general and oral health. These findings were found in bivariate as well as in multivariate analyses taking into account age, gender and lifestyle factors important for health. However, income and the ability of obtaining a given sum of money in one week, were the SEP measures that showed the strongest associations with the two health outcomes.

The main objective of the study was to investigate gradients in self-reported health relative to different socioeconomic measures and to evaluate the strength of the associations depending upon the influence of other important risk factors for poor health. Over the past three to four decades, the scientific literature has shown typical strong correlations between health—both self-reported and objective health measures—and a plethora of indices capturing socioeconomic status [1, 5, 8]. The WHO has reported on socioeconomic inequalities and health, and Marmot and Wilkinson were among the leading researchers to show empirically the importance of social health determinants [16, 17]. The eye-opener, however, was published in 1991 by Marmot and co-workers, describing health inequalities among British civil servants in the Whitehall studies [18]. There, the authors clearly revealed gradients of health in relation to the civil servant’s job position. In Sweden and elsewhere, several reports show parallel results of health inequalities based on social standing. However, few publications discuss the steepness of the gradients and similarities between self-reported general and oral health in relation to different socioeconomic measures. Thus, the findings of this study contribute to an overwhelming body of knowledge regarding health status and socioeconomic position. All four socioeconomic indices used in this study showed almost the same hierarchical structure in self-reported general as well as oral health. However, the strength of the risk measure varied with the outcome, where general health had stronger associations with all four SEP indices. These findings are similar to what Sabbah et al. [5] found with data from the NHANES study in the US. The analysis included income and education, and the pattern of the gradients and the strength of the relationships in that study and the present study are very similar, despite the data coming from different countries.

The findings from this study indicate that the four different SEP variables have similar patterns with regard to gradients in the ORs. However, the variables most clearly associated with material resources in a short-term perspective; i.e., income and ability to obtain a large sum of money in one week, showed the strongest risk ratios with respect both to general and oral health. This result may be indicative of a previously revealed association between financial resources and health status [10]. It could be argued that such a correlation would be expected for dental care, due to a high out-of-pocket payment system for adult dental care in most countries, and would apply also to Sweden, despite a national dental care insurance scheme. The Swedish national insurance scheme implies that dental care for children and adolescents is free of charge, but for adults the insurance is also universal but less generous. Perhaps more surprisingly, an equally strong association was found between poor general health and poor financial resources. Sweden has a more generous national insurance system for health care than for dental care, for which reason the individuals’ financial situation should be less important for their general health status. Other and more complicated risk and protective factors for general health may thus be required to explain our findings.

In the statistical modelling, we reduced the number of categories in some of the SEP variables, such as education and the ability to obtain money in one week, due to few responses in some categories. However, when all the categories were used in descriptive tables, the differences in self-reported poor general and oral health still revealed the same gradient pattern. For example, in the education variable, individuals with masters or PhD degrees were in the highest category and there were significantly fewer participants reporting poor health in this category, also compared with individuals with a university degree but without a research degree. Similar results have been reported by Robert Erikson [19], where the author found a clear gradient in age-related mortality and level of education. Individuals with a research degree were significantly more likely to live longer than individuals with lower levels of education, including people with professional degrees, such as doctors and lawyers.

This survey comprising a random sample of adult Swedish individuals has some weaknesses and strengths. The proportion of non-respondents was high (50.3%), and the mean age, proportion of women and Swedish-born in the sample was significantly higher compared with the official Swedish data. It may be argued that the steepness of the gradients and the strength of the odds ratios would be underestimated, as has been revealed in other large-scale epidemiological surveys in Sweden [10]. Thus, among non-participants, the proportions of socially disadvantaged people may also be higher compared with the participants. A weakness concerning the household income variable may be that the analysis did not take into account the number of individuals per household. Moreover, the exclusion of individuals not speaking Swedish from the study may be one important reason why fewer foreign-born participated in the survey. Another issue to take into consideration regarding the education variable is that young adults may not have reached their highest educational level at the time of the interview. The study design was observational and cross-sectional, why only associations, not cause and effect, can be established. Moreover, the outcome measure was self-reported health and no objective health status was included. However, the design involved a large number of randomly chosen adult individuals living in Sweden, and the variables included, particularly the SEP and perceived health variables, are widely used in the scientific literature.

## Conclusions

In conclusion, the results of this study show that: (i) there are socioeconomic gradients in self-reported health, both general and oral, irrespective of different socioeconomic measures; (ii) the slope of the odds ratios is similar for general and oral health and the presented socioeconomic measures, and (iii) there are significant and important differences in perceived health among adult individuals living in Sweden. The obvious interpretation of these results is that action is needed for better health and health care through upstream and downstream activities, both prevention and promotion, to ensure greater equity of health.

## Abbreviation

SEP: Socioeconomic position
